# Primary School Teachers’ Assessment Profiles in Mathematics Education

**DOI:** 10.1371/journal.pone.0086817

**Published:** 2014-01-23

**Authors:** Michiel Veldhuis, Marja van den Heuvel-Panhuizen

**Affiliations:** 1 Freudenthal Institute for Science and Mathematics Education, Utrecht University, Utrecht, the Netherlands; 2 Freudenthal Institute for Science and Mathematics Education & Faculty of Social and Behavioural Sciences, Utrecht University, Utrecht, the Netherlands; University of Westminster, United Kingdom

## Abstract

The aim of this study was to contribute to knowledge about classroom assessment by identifying profiles of teachers’ assessment of their students’ understanding of mathematics. For carrying out this study we used data of a nationwide teacher survey (*N* = 960) in the Netherlands. The data were collected by an online questionnaire. Through exploratory factor analyses the underlying structure of what is measured by this questionnaire was uncovered as consisting of five factors: Goal centeredness of assessment, Authentic nature of assessment, Perceived usefulness of assessment, Diversity of assessment problem format, and Allocated importance of assessing skills and knowledge. By using a latent class analysis four different assessment profiles of teachers were identified: Enthusiastic assessors, Mainstream assessors, Non-enthusiastic assessors, and Alternative assessors. The findings suggest that teachers with particular assessment profiles have qualitatively different assessment practices. The paper concludes with discussing theoretical implications of these assessment profiles and indications these profiles can offer both for designing material for professional development in classroom assessment and for evaluating changes in teachers’ classroom assessment practice.

## Introduction

Classroom assessment is crucial for students’ learning [1]. A main reason for this is that through classroom assessment teachers can gather information on their students’ skills and level of understanding to make decisions about further instruction. Based on this information teachers can adapt their teaching to their students’ needs and create an ideal learning environment for them in their classroom. Therefore, the use of classroom assessment as an integrative part of education has been named as one of the most important activities for teachers to improve student achievement (e.g., [2]).

Consequently, gaining knowledge about classroom assessment has high priority in educational research. The better we know how the individual teacher carries out the collection of data on students’ learning, the more we are able to optimize this process. Contributing to this knowledge was the aim of this study. Our focus was on classroom assessment in primary school mathematics education.

To realize this aim we built on a previous study which investigated how primary school teachers in the Netherlands collect information about their students’ progress in mathematics (see [3]). The data for this earlier study were collected by means of an online questionnaire. The prior analysis of these data gave a general overview of how often Dutch primary school teachers are using particular assessment methods, the purposes they are assessing for, and the teachers’ perceived usefulness of these assessment methods, and the relations between assessment methods, purposes, and perceived usefulness. In addition to these overall findings, the present study was aimed at gaining knowledge of how the assessment practices of individual teachers can be characterized within the universe of assessment skills and activities. In fact, in this study, we wanted to understand assessment from the conglomerate of choices a single teacher is making when collecting information about his or her students’ learning process. To achieve this we performed a secondary analysis of the earlier gathered questionnaire data to identify a profile characterization of every teacher’s assessment practice. The rationale for distinguishing assessment profiles of teachers is that these can contribute to our theoretical understanding of assessment as it is carried out by teachers. In addition, knowledge about these assessment profiles can help us in a practical sense with designing tailor-made courses for professional development that fit the teachers’ needs. Furthermore, these assessment profiles can provide us with a tool to measure changes in teachers’ classroom assessment practice.

### Theoretical Background: A Classroom Assessment Theory?

A scientific theory of any given process generally consists of a description of the constituting components, the causal mechanisms that govern these components, information about factors influencing all of these, and implications for practice. In the end, for further theory building, it is necessary that observational consequences of a theory are tested.

With respect to classroom assessment in mathematics education, many scholars have proposed tentative theories of classroom assessment. As such a variety of conceptualizations exists of what assessment in mathematics education is, and entails, which have abundantly been investigated and discussed. Generally, the skills teachers need to have in order to perform various assessment activities are part of these conceptualizations. Some go a bit further and also describe conceptual models integrating theoretical concepts and practices. However, the descriptions rarely surpass a mere listing of concepts related to assessment. In any case, testing a proposed theory about assessment is certainly not something that is frequently done.

To illustrate the great variety of approaches and methods describing teachers’ specific assessment skills and activities, and, more generally, models of assessment, we give a brief sketch of the available research (also strikingly labelled as a “patchwork” of research [4]). We start by describing research into the *assessment skills* of teachers (also called assessment literacy [5]), then we focus on inventories of teachers’ *assessment activities*, and finally we set out some *conceptual models of assessment* that outline relations between concepts, skills, and activities.

This sketch is structured following the recent change in focus in research and theories about classroom assessment: from descriptions of assessment skills teachers should have to teachers’ actual assessment activities. These two aspects of classroom assessment are evidently related, in the sense that the assessment skills a teacher has (or does not have) influence the assessment activities he or she actually uses in the classroom. Quite logically one could expect that there is a temporal, and maybe even a causal, link between assessment skills and assessment activities: if a teacher is not knowledgeable about assessment, he or she will probably not use assessment in the proper way. Both assessment skills and assessment activities have quite extensively been studied, and are used as a basis for concepts and conceptual models in theory on classroom assessment.

### Assessment Skills of Teachers

In the early 1990s the assessment skills of teachers became the main focus of assessment-related research. Ever since the publication of the first version of the standards for teacher competence in educational assessment of students [6], assessment skills have regularly been investigated [5], [7]–[10]. These standards were developed by an expert group based on a review of research literature focused on improving and defining the assessment skills teachers should have. The particular skills teachers were supposed to have according to these standards were (i) choosing and developing assessment methods, (ii) using assessment results for decision making and grading, (iii) communicating assessment results, and (iv) recognizing unethical assessment practices. These standards were clearly centered on teachers’ assessment competence, i.e. assessment skills, but made no mention at all of their actual assessment activities.

Brookhart [11] recently updated the standards for assessment, taking into account the recent surge the use of formative assessment has taken, especially after the influential work of the Assessment Reform Group [12] and the famous review study by Black and Wiliam [2], [13]. In the updated standards some assessment skills are still mentioned but the assessment activities of teachers such as setting goals, communicating learning intentions, and interpreting assessment results are given much more importance [11]. The same trend can be observed in the writings of the American National Board for Professional Teaching Standards (NBPTS [14]), where assessment practice is one of the certification standards:

Accomplished mathematics teachers *integrate a range of assessment methods* into their instruction to promote the learning of all students by designing, selecting, and ethically *employing assessments* that align with educational goals. They provide opportunities for students to reflect on their strengths and weaknesses in order to revise, support, and extend their individual performance (p. 61) *[emphasis added]*.

A combination of assessment skills and assessment activities is clearly advocated in the recent standards of both Brookhart [11] and the NBPTS [14]. The focus in the original version of their standards from over 20 years ago was exclusively on the *assessment skills* teachers should have, whereas in their more recent standards the *assessment activities* of teachers have become the focal point. This transfer can be seen as a parallel to the move from teacher-centered to student-centered education, in the sense that assessment skills only address the teacher, while assessment activities immediately imply that students are involved, in the sense of an interaction between teacher and students.

### Assessment Activities of Teachers

Descriptions of teachers’ assessment activities come in different forms and with manifold foci. Here we will outline some examples from research to illustrate the recurring types of assessment activities teachers are using. Most research on assessment activities has been done through a combination of surveys and classroom observations. For instance, McMillan [15], [16] inventoried the assessment activities of primary and secondary education teachers in the U.S., focusing on the information they used to grade their students’ performance. Here the assessment activity can be identified as collecting information and providing feedback through grading. Mavrommatis [17] used a framework based on interviews and observations to describe mathematics teachers’ assessment process, taking place in four phases, including evidence collection, evidence interpretation, teachers’ responses, and students’ reactions. For every phase the actions are described that teachers can undertake, for instance the type of questions they can use to elicit evidence of learning. Here the activities of assessment are observation and questioning to gather ‘evidence’ or information, and providing feedback to the students. A further example is the study by Wiliam, Lee, Harrison, and Black [18] on the effects of a professional development track in assessment for learning, where teachers had to use among others questioning and providing feedback. From the foregoing examples of research on teachers’ assessment activities (see also, [19], [20]) the following core activities of teachers’ classroom assessment practice, emanate: questioning, observation, and providing feedback.

In addition to capturing assessment activities, research has also portrayed the beliefs teachers have about assessment. These beliefs of teachers are chiefly related to the practical (activities) side of assessment. For example, teachers can conceptualize assessment as consisting of rich questioning, and providing feedback to move learning forward [21]. Furthermore, another way researchers have looked into the matter of assessment is investigating the relation between the core assessment activities, teachers’ assessment skills, and theories of learning and motivation. Then, we come close to what can be considered conceptual models of assessment.

### Conceptual Models of Assessment

As Brookhart [4] described in a review of research literature on classroom assessment, there are different approaches to study this topic:

Theory relevant to studying classroom assessment comes from several different areas: the study of individual differences (e.g. educational psychology, theories of learning and motivation), the study of groups (e.g. social learning theory, sociology) and the study of measurement (e.g. validity and reliability theory, formative and summative assessment) (p. 429).

This rich variety of perspectives from which assessment can be approached results in conceptual models about classroom assessment showing many different emphases (see [4]). Some authors mainly focus on feedback [22] or motivation through self-regulation [23], while others concentrate on scaffolding [24], for instance.

In addition some broader models have been described that include several factors determining classroom assessment. For example, McMillan [25] presented a model including teacher knowledge, external factors, and the realities teachers encounter in classroom as the most important influences on the instructional decision-making rationale, which in turn determine the classroom assessment practice. The classroom assessment practice ranged from quizzes and tests, to informal observation, which we can again identify as several of the assessment activities. Another broad vision of classroom assessment was provided by Watson [26] who listed concepts ranging from theoretical, such as psychological, cognitive, and social factors, via views of mathematics, interpersonal relations, attitudes, feedback or motivation, to classroom practice such as exercises, use of specific tasks for assessment, and homework. Similar to McMillan’s [25] model, again core assessment activities, assessment skills, and relations between them can be identified. In both models there is a whole system that exists around an individual learner and the assessor, which can be considered of great importance for assessment.

Yet, further models have been proposed as well. For example, Schneider and Gowan [27] suggested a ‘theory of action’ of formative classroom assessment. Four assumptions formed the basis of this model and in these assumptions we can once more identify assessment skills as well as activities. The first assumption in this model is the gathering of accurate information about student learning, the second is the analysis of the responses and inferences about learning, the third is providing feedback or adapting instruction, and the fourth is that the student uses this feedback to move forward. Black and Wiliam [28] proposed a framework for what they called the theory of formative assessment. This framework consisted of a description of practice for the teacher, learners, and peers during (formative) assessment. As a background for this framework they sketched relations between formative assessment and instruction-related issues such as cognitive acceleration, dynamic assessment, and models of self-regulated learning and classroom discourse. Finally, Pellegrino, Chudowsky, and Glaser [29] have also proposed a model of assessment that can be used to make the relations between different concepts more insightful. They used a triangle with on one end, the assessment activity of observation, the way to elicit evidence of students’ competences, and on the two other ends the assessment skills of interpretation, which refer to the process of making sense of the evidence, and the teacher’s model of students’ cognition or learning in the assessed domain.

A common denominator in all the foregoing models, frameworks, or attempts at theory building, is that they consider assessment to be an interactive process between students and teacher, where the teacher actively searches for information about students’ abilities and understanding (assessment activities), and communicates this with the students, as such giving them cognitive and motivational support (assessment skills) to offer learning opportunities. In the end, most studies focus on the purpose of assessment being the improvement of learning [30], [31]. Some researchers [22] have called the identification of the gap between the actual current level of performance and the aimed-for level the main goal of assessment. Furthermore, what can be concluded from these theoretical considerations on classroom assessment is that most are made up of a flat description of the relations between the core assessment activities and theoretical factors influencing assessment. Core assessment activities of questioning, observation, and feedback, that could be considered as part of contingent teaching [32] and links to psychological theories on motivation through feedback or self-regulated learning, recur in these considerations. Questioning is considered a mix of questions aimed at revealing what a student knows and questions that help a student to learn [20]. Similarly, the feedback teachers provide is generally formatively used and aimed at helping students acquire more knowledge, confidence, and understanding [33].

Although the aforementioned lists of assessment skills, activities, and conceptual models cannot be considered a fully-fledged, crystallized theory about assessment, they clearly illustrate that classroom assessment is a complex, all-encompassing process that fulfills a central role in instruction.

### Present Study

In our current investigation we followed the described trend from *assessment skills* teachers have, to *assessment activities*, focusing on what teachers report doing in their classrooms. The goal of the present study was the identification of teachers’ assessment profiles on the basis of questionnaire data on teachers’ reported assessment practice. Via these profiles we intended to characterize individual teachers’ assessment practice. Moreover we strived for a contribution to a better theoretical understanding of the assessment by teachers through the detection of relevant concepts in classroom assessment in mathematics education. We did not have the pretention to propose a new theory or model of assessment, but merely tried to identify clusters of factors in classroom assessment that are important for determining teachers’ assessment practice. The idea in this study was to go one small step further than just list concepts and their interrelations, and describe the factors that lie in between. The aim of the study was offering teachers and researchers of assessment in mathematics education a characterization of assessment practice through the determination of teacher profiles. The research question that guided this endeavor was: Can teachers’ current practice of assessment in primary school mathematics education be described by means of assessment profiles?

## Method

### Ethics Statement

Before starting to fill in the questionnaire teachers were provided with information on the researchers, on the purpose, and on the content of the research. Teachers were also given the choice to participate by agreeing to this information, or to not participate, and could quit the questionnaire at any moment. As all participants voluntarily subscribed to the study and data were analyzed anonymously, we did not formally ask teachers for written consent. Our research was on normal educational practice and we did not consult with an institutional review board (our institute which only focuses on educational research does not have such a board). All this is in line with section 3.4.1 of the VSNU (Dutch Association of Universities) regulations on the use of personal information in scientific research in the Netherlands, the Federal Policy for the Protection of Human Subjects of the National Science Foundation in the USA and section 8.05 “Dispensing with Informed Consent for Research” of the APA ethical standards.

### Online Teacher Questionnaire

An expert group consisting of researchers, test developers, education developers, measurement specialists, and didactical experts developed an online questionnaire to collect information on primary school teachers’ assessment practices and beliefs about assessment in mathematics [3]. This questionnaire contained 40 items (see Table 1 to 5), pertaining to the teachers’ (i) background characteristics, (ii) mathematics teaching practice, (iii) assessment practice, and (iv) perceived usefulness of assessment. Questions with different formats were included: fixed-response and items with a rating scale, but also some open-ended items. Lists of possible assessment methods, and purposes of assessment, were deduced from literature on classroom assessment [2], [13], [17], [34].

**Table 1:** Factor loadings of Goal centeredness of assessment.

Questionnaire item	Factor loading
Assessment purpose: Determine mastery	.793
Assessment purpose: Adapt instruction	.778
Assessment purpose: Determine progress	.734
Assessment purpose: Tune the speed of instruction	.728
Assessment purpose: Select mathematics subjects	.636
Assessment purpose: Investigate reasons for errors	.592
Assessment purpose: Formulate learning goals	.520
Assessment purpose: Provide feedback	.512
Assessment purpose: Establish level groups	.489
Assessment purpose: Stimulate thinking	.487
Assessment method: Textbook tests	.401
Assessment purpose: Stimulate use of scrap paper	.381
Frequency of need for assessment information	.374
Setting of clear goals for students	.363
Assessment method: Correct written work	.339
Assessment method: Questioning	.328
Assessment method: Observation	.301
*Cronbach’s alpha = .804*	

**Table 2:** Factor loadings of Authentic nature of assessment.

Questionnaire item	Factor loading
Assessment method: Practical assignments	.706
Assessment method: Teacher-developed tests	.643
Assessment method: Student-developed tests	.382
Importance of assessing: Students’ design skills	.322
Importance of assessing: Students’ memory skills	−.246
Assessment purpose: Assessing use of scrap paper	−.334
Importance of assessing: Students’ factual knowledge	−.353
Assessment method: Student monitoring tests	−.361
Assessment method: Correcting written work	−.378
Assessment method: Textbook tests	−.483
*Cronbach’s alpha:.456*	

**Table 3:** Factor loadings of Perceived usefulness of assessment.

Questionnaire item	Factor loading
Assessment can determine what students have learned	.880
Assessment results predict students’ performances	.851
Assessment helps to improve my teaching	.838
Assessment helps students to learn	.837
Assessment provides information about learning needs	.833
Assessment can be used to map strong/weak sides	.817
Assessment has much influence on my teaching^a^	.816
Assessment creates a better learning climate	.813
Assessment is an interruption of my teaching^a^	.800
Assessment informs what students can^a^	.760
*Cronbach’s alpha = .803*	
^a^These statements were originally phrased negatively in the questionnaire, e.g. “Assessment has *little* influence on my teaching”, and have been recoded	

**Table 4:** Factor loadings of Diversity of assessment problem format.

Questionnaire item	Factor loading
Mathematical problems in context	.930
Bare mathematical problems	.887
Mathematical problems where students explain their calculations	.875
Mathematical problems with more than one correct answer	.699
*Cronbach’s alpha = .770*	

**Table 5:** Factor loadings of Allocated importance of assessing type of skills and knowledge.

Questionnaire item	Factor loading
Importance of assessing procedural knowledge	.709
Importance of assessing factual knowledge	.707
Importance of assessing conceptual knowledge	.701
Importance of assessing memory skills	.684
Importance of assessing understanding skills	.675
Importance of assessing applying skills	.640
Importance of assessing analyzing skills	.631
Importance of assessing evaluation skills	.520
Importance of assessing self-knowledge	.473
Importance of assessing design skills	.425
*Cronbach’s alpha = .823*	

### Procedure of Data Collection

The sample of participating teachers was obtained through an open invitation by e-mail, which was sent successfully to 5094 primary schools for regular education in the Netherlands. Teachers who were willing to respond to the online questionnaire were promised a set of digital mathematical exercise material as a reward. In February 2012, we sent a renewed request to all teachers that did not fill in the questionnaire after the first request. The final sample included 960 teachers from 557 different schools, who filled in at least one question about their assessment practice. Of the sample of teachers 83.7% were female, and the mean age was 41.4 years (*SD = *11.6).

To investigate the representativeness of the sample we compared background characteristics with available national statistics [35]. Almost all variables, including age, gender, geographical location of the school, urbanization level of the school, textbook use, education, religious denomination of the school, and the size of the appointment of the teacher followed approximately the same distribution as the national statistics. See Supplementary material (File S1) for more details.

### Data Analyses

We analyzed the data in two steps. First, we looked into the factorial structure of the questionnaire and the underlying classes of teachers. Then, we investigated the differences between different classes of teachers on the factors of the assessment questionnaire. To identify the latent structure of what was measured by the questionnaire and be able to construct assessment profiles of teachers we used a combination of latent variable modeling techniques. In this approach it is important to be knowledgeable of the fact that every model is an oversimplification of reality, and can thus never be a perfect fit to the data. Additionally, no golden rules for deciding upon the fit of the model to the data exist; therefore we have to investigate the relative fit of the model in comparison to other, comparable, models. Then, to decide which model is most appropriate in describing the data it is advised to use substantive as well as statistical model fit checking [36]. Substantive model checking concerns checking whether the model’s predictions and constituents are in line with theoretical and practical expectations. Statistical model fit checking can be done in a variety of ways. There exists a multitude of statistical methods to compare the statistical merit of different models that can generally be divided in two categories. One is a statistical test of model fit, where the model of interest is compared via a likelihood ratio test or a χ^2^-test to neighboring models. The other is to compare statistical indicators such as information criteria or entropy between different nested models [37], [38].

In evaluating the different latent variable models in this study both the aforementioned statistical and the substantive model fit checking methods have been used. To explore the underlying structure of the items that measure teachers’ mathematics assessment practice, we performed several exploratory factor analyses, which was deemed most appropriate [39], because the questionnaire was constructed to measure assessment practice in mathematics education in a rather open way and no specific theoretical ideas about the factorial structure were proposed in advance. The technique of exploratory factor analysis was used to understand the structure of variation on measured variables by estimating the correlations between latent factors and these measured variables. Experts in factor analytical research have different opinions about which statistics to include to evaluate statistical model-data fit, but they generally agree that at least a χ^2^-statistic, the root mean square error of approximation (RMSEA), and the comparative fit index (CFI) should be reported [40]–[42]. To indicate acceptable to good model fit, the conventions are that the RMSEA should be around 0.06 [43] and the CFI more than 0.96 [44]. Using Mplus 5.21 [45] we performed exploratory factor analyses with weighted least squares method (WLSM) estimation and geomin oblique rotation. Finally, we took into consideration whether the items making up the factors had sufficiently in common and whether the factors theoretically made sense, which provided us with substantive reasons to decide upon fit and allowing us to name the factors accordingly.

Furthermore, to investigate whether these latent factors could also be used to interpret classes of teachers, we performed a latent class analysis. This is a statistical technique permitting the identification of underlying classes of individuals based on differences in their responses on items in a questionnaire or test. The underlying classes are identified on a discrete latent variable and permit the division of the sample in qualitatively differing subgroups [46]. As input for this analysis the item scores on the part of the questionnaire related to teachers’ assessment practice were used. The teachers in our sample were assigned to the different latent classes – that we will call assessment profiles – through modal assignment, i.e. they were assigned to the latent class to which they had the highest probability of belonging.

The differences between teachers with different assessment profiles on several background variables were investigated with analyses of variance, Kruskal Wallis and χ^2^-difference tests. Through these analyses we could determine the characterizing elements for every profile. All inferential analyses were performed in SPSS 20 [47] and the latent variable modeling was done in Mplus 5.21 [45].

## Results

### Teachers’ Assessment Practice

The earlier study in which we carried out a descriptive analysis of the questionnaire data [3] revealed that the Dutch primary school teachers involved in the survey used a mix of observation- and instrument-based methods in mathematics education. The most used observation-based methods were questioning, observing, and correcting written work (>77% weekly). The main instrument-based methods were textbook and pupil monitoring system tests (>85% several times a year). Teachers also used these methods for a mix of summative, formative, and diagnostic purposes. The most used purposes were: of the summative type, selecting what mathematics subjects should be taught (42% weekly); of the formative type, providing feedback, determining the speed of teaching, and adapting instruction (>62% weekly); of the diagnostic type, investigating reasons for errors (60% weekly).

### Teachers’ Assessment Profiles

After comparing one- to seven-factor solutions and eliminating items with cross loadings over |0.4|, an exploratory factor analysis delivered a five-factor solution that had a good enough fit (χ^2^(1076, N = 960) = 5494.1, *p*<.001, RMSEA = .064, CFI = .961). Also, these five factors all had eigenvalues over 2.5 and the scree plot showed a clear “elbow” after the fifth factor. The χ^ 2^-statistic of the overall model fit was significant, indicating a less than optimal fitting model. Nevertheless, this nested five-factor solution fitted significantly better than the four-factor solution, as illustrated by the Satorra-Bentler scaled χ^2^-test, which is unaffected by non-normality (*TRd*(df = 48) = 952.68, *p*<.0001). The different subscales used in the questionnaire loaded on these latent factors (see Tables 1–5 for the items constituting the factors and the corresponding scale’s Cronbach’s alpha), providing substantive evidence for this five-factor solution.

Regarding the items that constitute these factors we decided on the following names: (1) *Goal centeredness of assessment*, (2) *Authentic nature of assessment*, (3) *Perceived usefulness of assessment*, (4) *Diversity of assessment problem format*, and (5) *Allocated importance of assessing skills and knowledge*. Among the items in the factor *Goal centeredness of* assessment were whether teachers set goals for students and in particular the types of purposes their assessments served. The items relating to the type of exercises teachers included in mathematics tests made up the *Diversity of assessment problem format* factor. The *Allocated importance of assessing skills and knowledge* factor constituted of items measuring the importance of assessing different skills and types of knowledge. The *Perceived usefulness of assessment* factor comprised the items with statements about assessment such as: assessment helps to improve my teaching. The *Authentic nature of assessment methods* factor consisted of items measuring the frequency of the use of authentic assessment methods, such as practical assignments, student- or teacher-developed tests, and items loading negatively on this factor, such as the use of student monitoring system tests or textbook tests, that are the opposite of authentic assessment methods.

Correlations between the five factors are displayed in Table 6. Inspecting these correlations shows that *Authentic nature of assessment* was moderately negatively correlated with all factors (−.301< *r*<−.127, all *p*s <.01) except for *Perceived usefulness of assessment* with which it is uncorrelated. This indicates that the *Authentic nature of assessment* factor is quite different from the other factors, which stands to reason if one inspects the items belonging to this factor and its reliability. The items in this factor are very diverse (cf. Table 4) and the reliability was low of α = .456; whereas the items in the other factors were much more uniform with high internal reliabilities of α >.77. All other factors were weakly to moderately positively correlated with each other (.069< *r* <.425, all *p*s <.05).

**Table 6:** Correlations among the factors from the exploratory factor analysis (*N*s >857).

Factors	GC	DAF	AA	IASK	PUA
GC. Goal centeredness of assessment	–				
DAF. Diversity of assessment problem format	.154**	–			
AA. Authentic nature of assessment	−.301**	−.127**	–		
IASK. Allocated importance of assessing	.346**	.102**	−.148**	–	
PUA. Perceived usefulness of assessment	.262**	.069*	.025	.425**	–

*Note.* These are Pearson’s *r* coefficients. **p<.01. *p<.05.

**Figure 1:**
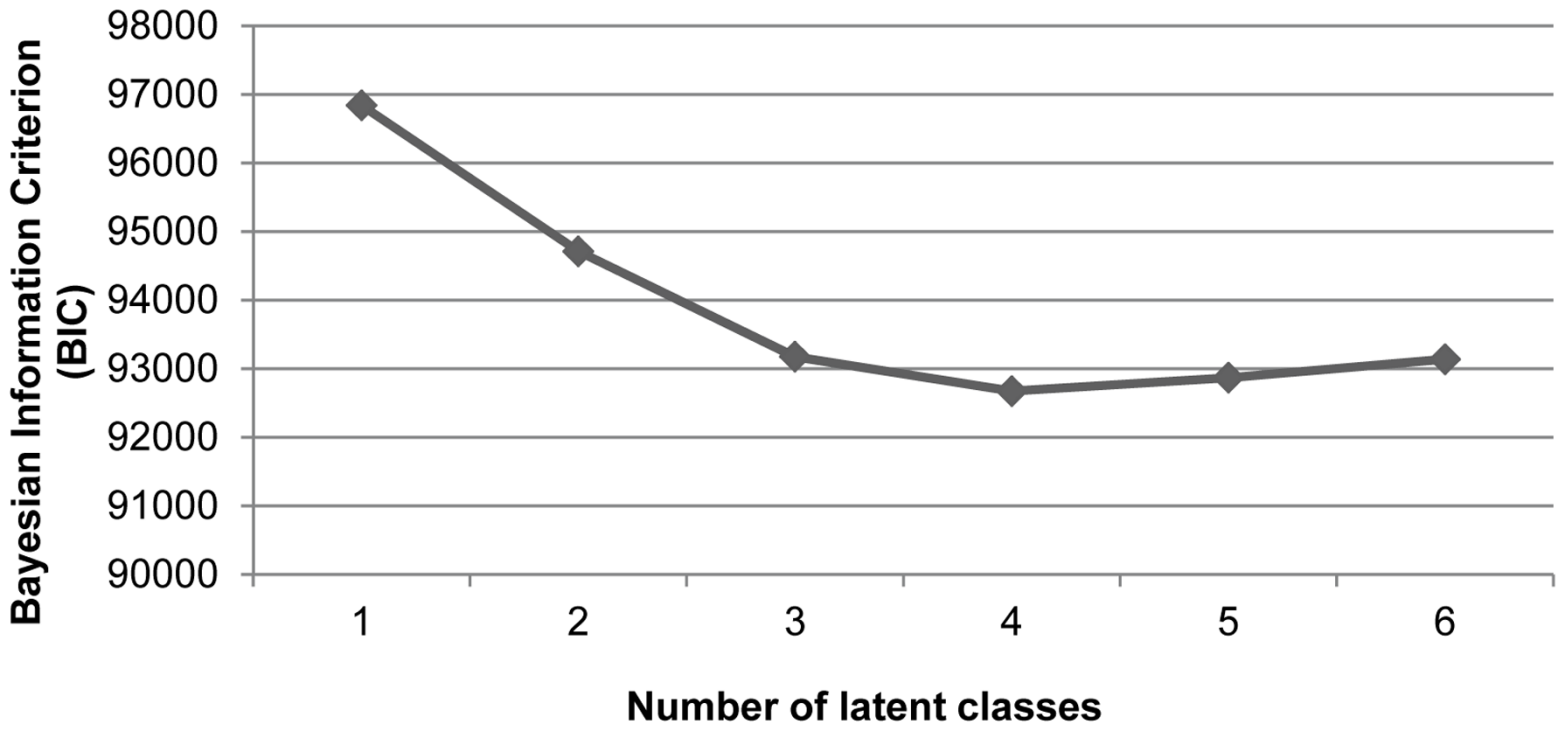
The value of the Bayesian Information Criterion for one to six latent classes.

The analysis carried out thus far gave us an approximation of the underlying structure of the questionnaire, but not yet information on teachers that could be used in practice. To be able to characterize teachers’ assessment practice and assign them to different assessment profiles we performed a latent class analysis using all variable scores as input. As such we were able to check whether we would be able to show differences between the latent classes on the factors we found separately. We used the Bayesian Information Criterion (BIC) and entropy to select the number of latent classes that best summarizes the variation data. As shown in Figure 1 the value of the BIC decreased until four latent classes and increased subsequently. This indicates that four latent classes provided the best fitting solution, as a lower value of the BIC indicates a better fit. The relative entropy of.93 (measuring the uncertainty of the classification, from 0 =  high uncertainty to 1 =  low uncertainty [48]) of the latent class model was high; indicating that the four classes were clearly separated [37]. Including age, gender, grade, or textbook use as covariates did not improve the fit of the model. Having four latent classes provided the best solution.

**Figure 2:**
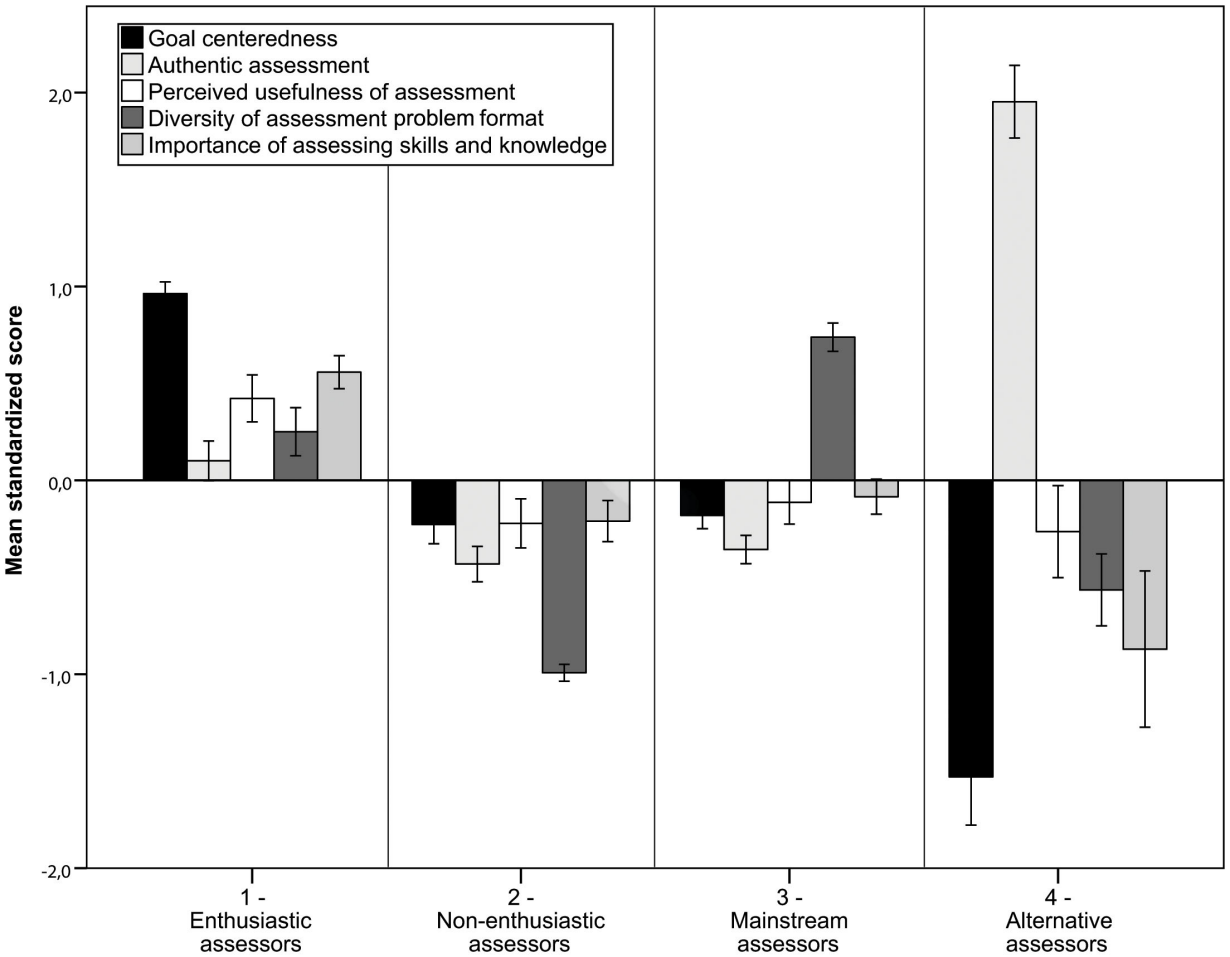
Mean standardized scores on factors for teachers in the four latent classes. *Note*. Whiskers indicate 95% confidence interval.

To find out whether teachers thus assigned to the four latent classes differed on the five factors of assessment identified before, we performed several analyses of variance. The results showed that teachers from one latent class to another differed significantly from each other. We found large effects for *Goal centeredness of assessment* (*F*(3, 852) = 324.2, *p<*.001, η_p_^2^ = .533) and *Diversity of assessment problem format* (*F*(3, 852) = 275.2, *p<*.0001, η_p_^2^ = .492), and medium to small effects for *Authentic nature of assessment* (*F*(3, 852) = 258.0, *p<*.001, η_p_^2^ = .476), *Allocated importance of assessing skills and knowledge* (*F*(3, 852) = 60.3, *p<*.001, η_p_^2^ = .175), and *Perceived usefulness of assessment* (*F*(3, 852) = 22.8, *p<*.001, η_p_^2^ = .074). Post-hoc tests using Bonferroni correction showed that the differences between all four latent classes were significant for *Diversity of assessment problem format* (all *p*s <.0001; see Figure 2 for the directions of these differences). Concerning the scores on *Goal centeredness of assessment* (*p = *1.00), *Authentic nature of assessment* (*p = *1.00), and *Allocated importance of assessing skills and knowledge* (*p = *.724), teachers in the second and third latent classes did not differ significantly from each other; differences between teachers in the first and fourth latent classes, however, were significant (all *p*s <.001). On *Perceived usefulness of assessment* teachers in the first latent class scored significantly higher than teachers from the other three classes (*p*<.001). Figure 2 shows the profiles of teachers from the four different classes in relation to the five standardized measures of teachers’ mathematics assessment practice.

Based on the results of these profile analyses we interpreted the different profiles as follows. In the first class, the teachers (28.5%) had above average scores on all assessment practice measures, with particularly high scores on *Goal centeredness of assessment*, *Perceived usefulness of assessment*, and *Allocated importance of assessing skills and knowledge*: they were aware of the different possibilities assessment offers them, reported using them likewise, and did this for a variety of goals. As such we considered these teachers to be *enthusiastic assessors*. Teachers in the second latent class were labelled as *non-enthusiastic assessors*. These teachers (25.8%) had scores below average on all measures, particularly on *Diversity of assessment problem format*. They viewed assessment more often in a negative way and used it accordingly less and in a less diverse way. Teachers in the third latent class were considered *mainstream assessors*. On four measures these teachers scored slightly below average, with the exception of the high score for the *Diversity of assessment problem format*. We called them *mainstream assessors,* because they scored generally close to average and most teachers belonged to this profile: 35.3% of our sample. Finally, the teachers from the fourth latent class (10.3%) were named *alternative assessors*. Teachers in this profile had an ambiguous view of assessment. On the one hand they reported a lot of *Authentic nature of assessment* use; for example, they devised their own tasks and tests. On the other hand they had scores below average on the remaining measures, with particularly low scores on *Goal centeredness* and *Allocated importance of assessing skills and knowledge*, clearly reflecting that they do not find assessment important, necessary, or helping them to reach certain goals.

### Teacher Characteristics and Assessment Profiles

To investigate which background characteristics are related to teachers’ attribution to one of the latent classes, we compared the scores for teachers with different profiles. In Table 7 the standardized means per profile for the five factors of the questionnaire, as well as the means on background variables, are displayed. With an analysis of variance we found that *non-enthusiastic assessors* (*M = *44.3, *SD = *11.5; *F*(3, 952) = 8.176, *p*<.001) were significantly older than *enthusiastic assessors* (*M = *40.8, *SD = *12.0; *p = *.003, *d = *0.30 (95% CI: [−0.71, 1.31])) and *mainstream assessors* (*M = *39.7, *SD = *11.1; *p*<.001, *d = *0.41 (95% CI: [−0.50, 1.32])). The number of years of teaching experience showed the same pattern (*F*(3,952) = 6.705, *p*<.001); which seems logical, as age and teaching experience correlate highly *r = *.830. *Enthusiastic assessors* (*M = *3.8, *SD = *1.2) worked significantly more days than *non-enthusiastic assessors* (*M = *3.5, *SD = *1.2; *F*(3, 949) = 2.873, *p* = .035, *d = *0.25 (95% CI: [0.15, 0.35])). Belonging to an assessment profile was significantly related to whether teachers obtained their professional qualification from a teacher education college for primary school teachers (χ^2^(3, *N* = 960) = 18.97, *p*<.001); proportionally few *alternative assessors* attended such a college (only 69% against 77–87% for the other profiles) The assessment profile was not significantly related (χ^2^(6, *N = *960) = 10.82, *p = *.094) to the type of pedagogical-didactical approach of the primary schools where the teachers were working – including regular schools and schools with a specific organization or teaching method such as Montessori and Dalton schools. Grade level and profile membership were significantly related (χ^2^(12, *N = *941) = 576.94, *p*<.001). *Alternative assessors* were mostly kindergarten teachers (80%), whereas there were very few (5%) in the other profiles. Proportionally, more *mainstream assessors* (53%) taught Grade 4 to Grade 6, than *enthusiastic* (45%) and *non-enthusiastic assessors* (50%). There was also a significant relation between gender and assessment profile (χ^2^(3, *N = *956) = 28.09, *p*<.001). Very few male teachers were *alternative assessors*; just 2%, whereas in the other profiles at least 13% of the teachers were male. The time teachers reported using to assess mathematics every week showed a pattern that reinforced the interpretation of the profiles. *Enthusiastic assessors* dedicated more time to the assessment of their students (*M = *85.61, *SD = *70.0) than in all three other profiles (*F*(3, 863) = 6.378, *p*<.001; post hoc Tukey all *p*s = .003). Analysis with a Kruskal Wallis test followed by a post-hoc Mann-Whitney test showed that *enthusiastic assessors* revised the level groups for their students with a higher frequency than teachers from the other profiles (χ^2^(3, *N = *955) = 57.98, *p*<.001), and *mainstream assessors* more often than *alternative assessors* (*p = *.03). Additionally, the frequency with which they discussed goals with students was higher for *enthusiastic assessors* than for *non-enthusiastic* and *mainstream assessors*, and all these frequencies were higher than for *alternative assessors* (χ^2^(3, *N = *951) = 104.91, *p*<.001). The need for assessment information was higher for *enthusiastic assessors*; they needed this more often than teachers from the other profiles (χ^2^(3, *N = *862) = 117.95, *p*<.001). *Alternative assessors* were different concerning the assessment methods they considered to be most relevant. They found practical assignments (χ^2^(3, *N = *883) = 170.74, *p*<.0001) and teacher-developed exercises (χ^2^(3, *N = *883) = 95.44, *p*<.001) considerably more relevant than teachers from the other profiles, and textbook tests (χ^2^(3, *N = *883) = 234.12, *p*<.001) and student monitoring system tests (χ^2^(3, *N = *883) = 32.47, *p*<.0001) less relevant. As a conclusion we summarized the main findings on the four different assessment profiles in Table 8.

**Table 7:** Mean values of factors constituting the profiles (above dotted line) and of related variables, and the significant profile differences.

	Total	Assessment profiles				Significant differences
		1. Enthusiastic	2. Non-enthusiastic	3. Mainstream	4. Alternative	
Goal centeredness (z)	–	**0.96**	−0.23	−0.18	0.01	1>4>2, 3
Diversity of assessment problem format (z)	–	0.25	−0.99	**0.74**	−0.57	3>1>4>2
Authentic nature of assessment (z)	–	0.10	−0.43	−0.36	**1.95**	4>1>2, 3
Allocated importance (z)	–	**0.56**	−0.21	−0.08	−0.87	1>2, 3>4
Perceived usefulness (z)	–	**0.42**	−0.22	−0.11	−0.26	1>2, 3, 4
Age (in years)	41.4	40.8	**44.4**	39.7	41.3	2>1, 3
Gender (% male)	16	13	23	18	**2**	4<1, 2, 3
Teaching experience (in years)	16.2	15.3	**18.8**	15	15.8	2>1, 3
Teacher trainer college (%)	80	79	76	**86**	69	3>2, 4
Peda.-didactical approach (% regular)	84	81	88	84	77	n.s.
Students in class	23.8	23.8	23.4	24	23.5	n.s.
Professional development sessions	10.2	11.7	9.9	8.8	11.6	n.s.
Size of position (days/week)	3.7	3.8	3.5	3.7	3.6	1>2
Time for assessment (min/week)	72.2	**85.6**	67.2	68.4	59.9	1>2, 3, 4
Frequency revision level groups^a^	3.2	**3.5**	3.2	3.1	3	1>2, 3, 4; 3>4
Frequency discuss goals^a^	4.3	**5.3**	3.9	4.1	3.2	1>2, 3>4
Frequency need information^a^	4.5	**5.2**	4.3	4.2	4	1>2, 3, 4; 2>4

*Note*. The significantly highest value per row is printed in **bold**.

a 1 =  *Rarely to never*, 2 =  *Yearly*, 3 =  *A few times a year*, 4 =  *Monthly*, 5 =  *Weekly*, 6 =  *A few times a week.*

**Table 8:** Summary and description of teachers’ assessment profiles.

Assessment profile 1: *Enthusiastic* *assessors* (28.5%)	Enthusiastic assessors had above average scores on all measures in the questionnaire: they were particularly goal-centered in assessment, and perceived it to be useful and important. Teachers with this profile dedicated more time to assessment than teachers with the other profiles.
Assessment profile 2: *Non-enthusiastic* *assessors* (25.8%)	Non-enthusiastic assessors had below average scores on all measures in the questionnaire: they did not think assessment to be important or useful, and particularly did not use a variety of problem formats to assess mathematics. Teachers with this profile were generally older than teachers with the other profiles.
Assessment profile 3: *Mainstream* *assessors* (35.3%)	Mainstream assessors scored slightly below average on most measures in the questionnaire: they were less goal centered, used less often authentic assessment, perceived assessment as averagely useful and important, but used more diverse problem formats to assess mathematics. Teachers with this profile were more often educated at a teacher education college for primary school teachers than teachers with the other profiles.
Assessment profile 4: *Alternative* *assessors* (10.3%)	Alternative assessors had very low scores on all measures, except on authentic nature of assessment: they were not goal centered, perceived assessment not as useful or important, and did not use a diversity of mathematics problems. Teachers with this profile were mostly kindergarten teachers, less often educated at a teacher education college for primary school teachers, almost exclusively female, and half of them did not use a textbook for mathematics.

## Discussion

In this study we have identified four distinct teacher profiles with clearly different scores on the five underlying factors from a mathematics assessment questionnaire. Exploratory factor analyses permitted to decide on the number and content of the underlying factors of the questionnaire, followed by a latent class analysis that determined the number of distinct latent classes to which individual teachers belonged. The assessment profile to which most teachers in our sample belonged was the *mainstream assessors* profile (see Table 8). In this profile most teachers regularly used different types of assessment, test-based and observation-based, for both summative and formative purposes. On all factors, i.e., *Goal centeredness of assessment, Diversity of assessment problem format, Summative assessment methods, Allocated importance of assessing skills and knowledge*, *Perceived usefulness of assessment,* and *Authentic nature of assessment methods,* teachers with this profile scored around the mean. The next biggest group was the *enthusiastic assessors*. Teachers with this profile were very aware of the different possibilities assessment offers them, and used them likewise. On all components these teachers scored above the mean, with a peak on *Goal centeredness of assessment*. An almost equally large group of teachers were the *non-enthusiastic assessors*. These teachers viewed assessment more often in a negative way and used it accordingly less. On all factors, teachers with this profile scored below average. Finally, there were the *alternative assessors*. Teachers with this profile had an ambiguous view of assessment. Although they reported a lot of own input in assessment and devised their own tasks and tests, they did not find assessment important or necessary. We found that most teachers with this profile were actually kindergarten teachers, which might explain their divergent profile: in kindergarten standardized assessment is almost absent from the classroom and as such seen as unnecessary.

In sum, we can say that our main aim of identifying meaningful assessment profiles has been achieved, but the question that remains is: How can this characterization contribute to the existing plethora of conceptualizations and lists of assessment activities and skills? Based on our analyses, we can conclude that the factors mainly fall under the headings of assessment activities (*Authentic assessment* and *Diversity of assessment problem format*) and assessment skills (*Goal centeredness of assessment*, *Perceived usefulness of assessment*, and *Allocated importance of assessing skills and knowledge*). The relations we have found between these factors and the characteristics of the teachers have enabled us to determine profiles with clear differences between teachers. These profiles serve a double purpose. First, they permit to typify the assessment teachers perform in their classroom, and as such they can be used to propose tailor-made professional development for teachers with specific profiles. A second purpose is that this profile characterization makes a connection between the assessment activities and assessment skills of teachers, and that this connection could be used in the further development of conceptualizations and eventually a theory of classroom assessment.

When using the results of our study it should be taken into account that the study is based on a rather large but local sample; all teachers came from the Netherlands. Moreover, the voluntary participation of the teachers in our study may have resulted in some bias in the sample. Although we found the teachers in our sample quite representative of the population of primary school teachers in the Netherlands, it is still possible that participating teachers were special in other aspects; they could, for instance, have been positively biased towards assessment in their responses on the questionnaire. The purpose of our survey, however, was rather neutral and by only asking teachers to inform us anonymously about their assessment practice, we think this potential positive bias did not have a detrimental influence on the reliability of teachers’ responses. The fact that we used self-report data from teachers as a basis for all analyses in this study could have led to another limitation. In the interpretation of our results it is important not to forget that we evidently cannot be entirely sure from these self-report data that teachers actually do, believe, and think what they report to be doing, believing, and thinking. Nonetheless, teachers had no reason whatsoever to misreport their behavior or opinions, because the questionnaire was anonymous. Yet, to control for this, in further research it would be interesting to compare and combine different sources of data about teachers’ practice in mathematics assessment, such as observations, interviews, and student data, and integrate these into the assessment profiles.

Taking supplementary sources into account and extending this study into classroom assessment in primary school mathematics education to other countries might lead to getting even more robust assessment profiles. Another approach would be to look into applications of the assessment profiles, for instance targeting a specific type of teachers for professional development. It would also be possible to investigate the effects of professional development on the assessment profile of teachers; teachers could move from one profile to another. Furthermore, another approach would be to link teachers through their profiles to levels of student performance; as assessment and instruction are intrinsically linked [49], different types of assessment would probably be linked to different learning results. In a sense this is in line with results of research on the effects of classroom assessment [2], [4], [12], [13], [17]–[21]; teachers that assess more and in an effective, often formative, fashion, have been shown to ensure more learning gain in their students. A tentative hypothesis would be to expect this to come from the teachers that are *enthusiastic assessors* for example, given that they assess often and use assessment in various ways (cf. Table 8).

To conclude, through our profile characterization of teachers’ assessment practice we were able to select some of the skills and activities from the universe of assessment skills and activities. In this way we have brought some structure to the many possible characterizations of assessment practice and skills that exist. These assessment profiles can contribute to a better theoretical understanding of classroom assessment and can also be useful in a practical manner as a basis for designing professional development and instruments for measuring teachers’ assessment practice.

## Supporting Information

File S1 **Representativeness of the sample.**
(DOCX)
