# Supplementary material for: Primary School Teachers’ Assessment Profiles in Mathematics Education
Source: PLoS One. 2014 Jan 23;9(1):e86817. doi: 10.1371/journal.pone.0086817 (PMC3900652; doi:10.1371/journal.pone.0086817)
Supplement: File S1 — Representativeness of the sample. (DOCX) [file pone.0086817.s001.docx]

**Supplementary material: representativeness**

To investigate the representativeness of the sample, as teachers from schools that did respond differ could be different from those that did not, we compared their background characteristics to the available national statistics (CBS, 2012; www.stamos.nl, 2010) of all primary school teachers in the Netherlands (NL). In our sample 83.6% of the teachers were female and 15.6% male (all primary school teachers in the Netherlands in 2010, NL: 85.5% female and 14.5% male) with a mean age of 41.8 years (*SD* = 11.66; NL: 43.4 years). The amount of experience in teaching years of these teachers followed the same bimodal distribution as in the total population of Dutch primary school teachers with a grand mean of 16.5 years. Of the teachers 68% worked part-time (most worked three or four days per week) and 31.9% worked fulltime. Most teachers (78.9%) were groomed at the PABO (teacher training school, see Table S1; in NL: 78.9%).

Table S1: Prior education of teachers in the sample

| **Prior education** | | **Per cent** | **N** |
| --- | --- | --- | --- |
| High school | VMBO (preparatory vocational education) | 16.4 | 202 |
|  | HAVO (Higher general secondary education) | 37.7 | 463 |
|  | VWO (Preparatory scientific education) | 10.4 | 128 |
| Vocational education | MBO (Intermediate vocational education) | 2.1 | 26 |
|  | KLOS (Kindergarten teacher training) | 5.6 | 69 |
| Higher vocational education | PABO (Teacher training) | 78.9 | 969 |
|  | HBO (Higher vocational education) | 14.3 | 175 |
| Scientific education | HBO masters | 4.6 | 56 |
|  | University | 4.6 | 57 |

The different textbooks teachers in our sample use are displayed in Figure S1.

Figure S1: Textbook use in our sample (lighter grey) and in the Netherlands (dark grey)

The teachers also provided some data about their schools. For example, whether they had a religious connotation (catholic: 29.6%, or protestant: 29.2%; in NL 29.7% and 29.8%) or not (35.8%; in NL 33.2%). Most schools (81.5%) did not have a special teaching philosophy, of the 17% that did, Dalton, Montessori, and Jenaplan were the most common (*Ns* > 20).

Figure S2: Proportion of schools per province in our sample (N = 1220) and in the Netherlands (N=6848)

The geographical distribution of the participating schools generally followed the national distribution as well (see Figure S2), nonetheless teachers from the province of Zuid-Holland were slightly overrepresented and those from Limburg underrepresented in the sample.

Figure S3: Percentages of urbanization levels around schools in our sample and in the Netherlands

The urbanization level, comparable to social economic status in neighborhoods, is displayed in Figure S3. Here as well, can be seen that the distributions generally looked alike, even though there were more teachers in the sample from strongly urbanized regions and less from rural regions in comparison to the national sample. As most of these indicators are almost identical from our sample to the national percentages, our sample, even though its teachers had not been selected randomly or in a stratified way, is quite representative of the population.
